# Separation and purification of nylon 54 salts from fermentation broth by an integrated process involving microfiltration, ultrafiltration, and ion exchange

**DOI:** 10.3389/fbioe.2024.1448927

**Published:** 2024-08-01

**Authors:** Xiaojie Zhao, Qixu Hu, Yue Yang, Jiao Feng, Xin Wang, Ganlu Li, Hui Li, Kequan Chen

**Affiliations:** State Key Laboratory of Materials-Oriented Chemical Engineering, College of Biotechnology and Pharmaceutical Engineering, Nanjing Tech University, Nanjing, Jiangsu, China

**Keywords:** fermentation broth separation, membrane separation, ion exchange resin, crystallization, nylon 54 salt

## Abstract

Nylon 54 is a novel, biodegradable polyamide with excellent thermal resistance and water absorption properties. It can be polymerized using bio-based cadaverine and succinic acid as monomers. Traditional separation methods isolate individual monomers from the fermentation broth through acidification or alkalization, resulting in significant amounts of waste salts; however, synchronous separation of dibasic acids and diamines has not been reported. This study investigated an integrated process for the separation and extraction of nylon 54 salts from a co-fermentation broth without acidification or alkalization. We meticulously optimized the operational parameters of the integrated process to achieve maximum separation efficiency. Following microfiltration, ultrafiltration, and decolorization, the bacterial eliminating rate was ≥99.83%, and the protein concentration was ≤40 mg/L. The absorbance of the decolorized solution was ≤0.021 at 430 nm, and the recovery rate of nylon 54 salt reached 97%. Then, the pretreated solution was passed through sequential chromatographic columns, which effectively removed organic acid by-products (such as acetic acid and lactic acid), SO_4_^2−^, and NH_4_^+^ from the fermentation broth, resulting in a cadaverine yield of 98.01% and a succinic acid yield of 89.35%. Finally, by concentrating and crystallizing the eluent, the simulated fermentation broth yielded nylon 54 salt with a purity of 99.16% and a recovery rate of 58%, and the real fermentation broth yielded nylon 54 salt with a purity of 98.10% and a recovery rate of 56.21%. This integrated process offers a sustainable and environmentally friendly pathway for the complete biosynthesis of nylon 54 salt and has the potential to be extended to the preparation of other nylon salts.

## 1 Induction

Nylon, also known as polyamide, is the most common engineering plastic with wide applications in textiles, (Anwar et al., 2020) synthetic fibers, (Weerappuliarachchi et al., 2020) electronics, (Palsaniya et al., 2020) and medical fields (Sun et al., 2023). Its main chain contains amide repeating units (−CO−NH−) and different numbers of carbon atoms, thus exhibiting various material properties (Pradeilles et al., 2020). Most monomers for nylon synthesis are derived from nonrenewable petroleum resources, and the production generates harmful chemicals and greenhouse gases, such as carbon dioxide, causing environmental pollution and exacerbating global warming. Unlike petroleum-based nylon, bio-based nylons (PA54, PA56, and PA1010) are polymerized by monomers (such as dibasic acids, diamines, and lactams) derived from renewable biomass (such as glucose, cellulose, and vegetable oils) through biotransformation and various physical and chemical processes. This green, low carbon, and sustainable production process has attracted increasing attention and demonstrated great development potential (Lee J. A. et al., 2019; Ko et al., 2020).

Bio-based nylon 54 can be synthesized from bio-based cadaverine and succinic acid and has excellent mechanical and processing properties, making it a new type of fully bio-based biodegradable polyamide (Lee et al., 2020). Recently, with the development of synthetic biology, the industrial production of succinic acid and cadaverine has been realized; therefore, bio-based nylon 54 is expected to partially replace nylon 66 as an alternative fiber and engineering plastic in the future.

Researchers have dedicated to producing key nylon precursors, such as succinic acid, butanediamine, cadaverine, and oxalic acid, from non-food biomass through synthetic biological microbial fermentation (Liu et al., 2022; Vardon et al., 2022; Liu et al., 2023; Feng et al., 2024). However, the process still faces many challenges in terms of economic viability and environmental friendliness; for example, large amounts of acid and alkali are required in the separation process, which generates abundant waste salts and increases separation cost. We co-produced cadaverine and succinic acid in *Escherichia coli* (Wang et al., 2018; Gao et al., 2022) allowing their direct separation from fermentation broth for polymerization. This separation process does not require acid or alkali adjustments, thus reducing waste generation and separation cost. However, direct separation and extraction of nylon 54 salt from the fermentation broth remains challenging because the broth contains high concentrations of microbial cells as well as organic by-products, such as acetic acid, lactic acid, organic amines, and some inorganic salt ions. These by-products not only affect the yield and purity of the product but also inhibit the polymerization reaction, leading to polymers with a low molecular weight and a low concentration. All components in the fermentation broth must be thoroughly separated from the nylon 54, which is difficult to achieve in a single separation step. Therefore, it is crucial to develop an efficient downstream integrated separation process to remove impurities produced by microbial metabolism.

Many techniques exist for separating and purifying products from fermentation broths, including membrane separation, ion exchange, distillation, extraction, and crystallization (Khalid et al., 2019; Li et al., 2022; Speer et al., 2023). Microfiltration (MF) and ultrafiltration (UF) membrane separations are based on pore size sieving mechanisms, in which the pore size determines the molecular weight cutoff of the membrane, whereas nanofiltration membrane separation primarily relies on steric hindrance and charge repulsion (Luo and Wan, 2023). Membrane separation processes are deemed economical because of their high separation efficiency and scalability. Many studies have reported the use of MF and UF membrane separation as pretreatment steps for post-fermentation solutions to remove large molecular weight substances, such as microorganisms, proteins, and pigments, from the fermentation broth (Braune et al., 2021; Li et al., 2021; Santos et al., 2024). However, microorganisms and large molecular proteins can adhere to the membrane and form a biomass layer (Qi et al., 2023), significantly reducing membrane flux and separation efficiency and sometimes requiring shutdowns for membrane cleaning. Such membrane fouling not only increases operational costs but also causes permanent damage to membrane performance (Wang et al., 2023) making the development of membrane cleaning processes crucial. Macroporous ion exchange resins, owing to their strong adsorption capacity, high selectivity, low cost, simple process, renewability, and environmentally friendly nature, have been used for separation and purification of amino acids (Schmitt et al., 2021), organic acids (Zhou et al., 2023), organic amines (Sun et al., 2018), pharmaceuticals (Jing et al., 2019), food (Can et al., 2024) and chemicals (Møller et al., 2024). Crystallization is a separation method with a long history of removing small amounts of impurities and obtaining high-quality products. It is widely used as the final step in the production of various chemical products and intermediates, including nylon 56 salt (Yang et al., 2020), nylon 66 salt (Kiyotsukuri et al., 1996) and pharmaceuticals (Tsarfati et al., 2021).

**FIGURE 1:**
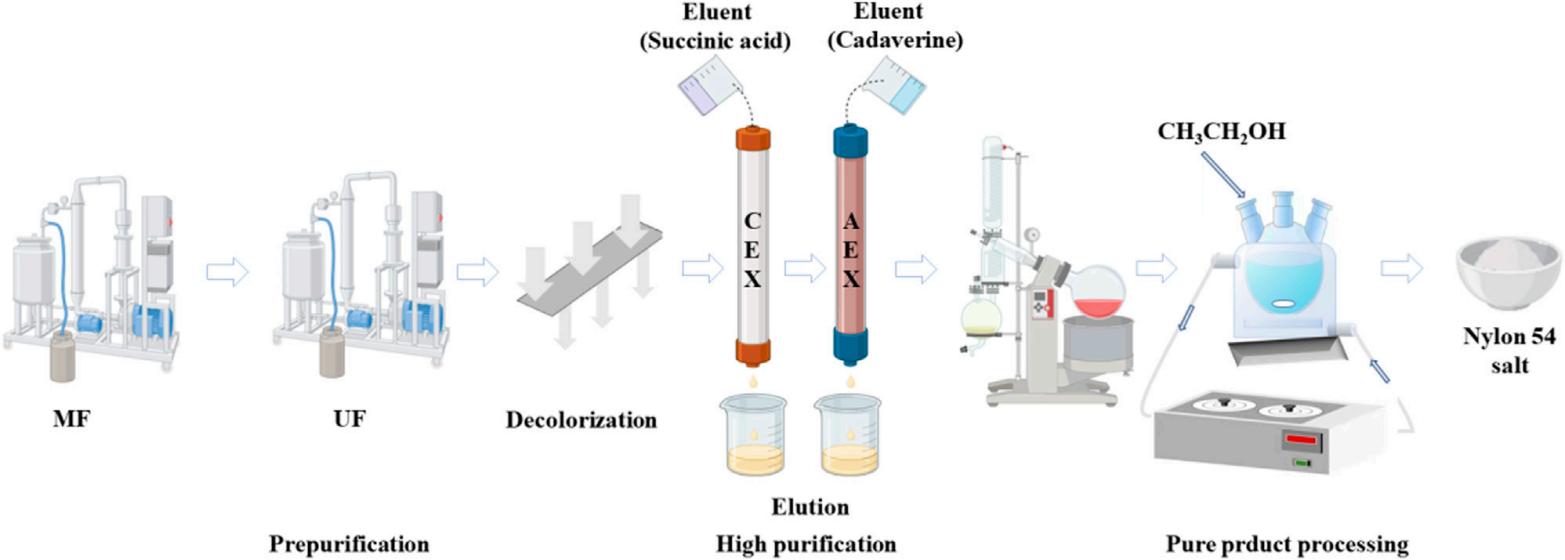
An integrated process for separating cadaverine and succinic acid from a co-fermentation broth.

In this study, we propose an integrated separation process consisting of four separation units (Figure 1): MF, UF, ion exchange, and crystallization to synchronously separate dibasic acids and diamines from the co-fermentation broth. In the pretreatment step, crossflow MF was used as a pretreatment to remove microorganisms and suspended colloidal particles. Subsequently, the broth was continuously recirculated in the feed tank to the UF separation process, where larger molecules and proteins were separated from the fermentation broth. Then, powdered wood-based activated carbon was used for decolorization, completing the pretreatment of the fermentation broth. In the second step, residual by-products and inorganic ions were removed from the mixed salt solution by ion exchange chromatography, and the adsorption mechanism of the resin was explored. Finally, the solution was evaporated and concentrated to obtain a highly concentrated nylon 54 salt solution, and high-purity nylon 54 salt crystals were obtained via crystallization. This paper proposes an integrated separation process with low operating cost, low energy consumption, and significantly lower waste salt production than traditional separation processes, thus providing a foundation for the industrial production of nylon 54 salt.

## 2 Materials and methods

### 2.1 Chemicals and reagents

Nylon 54 salt fermentation broth and deionized water were provided by our laboratory. Cadaverine and succinic acid were added to the simulated fermentation medium to prepare a synthetic solution for evaluating the separation of the nylon 54 salt. MF membrane (0.1 μm, polyethersulfone material, effective membrane area 0.32 m^2^) and UF membrane (5,000 D, polyethersulfone material, effective membrane area 0.32 m^2^) were purchased from Shanghai Kailabo Instrument Equipment Co., Ltd. Macroporous ionic exchange resins were purchased from Yuanye Biotechnology Co., Ltd. (Shanghai, China), which included weakly acidic cation exchange resins D113, D150, D152, and D155 (Table 1) and weakly basic anion exchange resins D311, D314, D315, and D318 (Table 2). Ethanol, hydrochloric acid, lactic acid, sodium hydroxide, acetonitrile, and trifluoroacetic acid were purchased from Aladdin (Shanghai, China).

**TABLE 1:** Parameters of cation exchange resins.

Resin	Matrix	Particle size (mm) (%)	Exchange capacity (mmol g^−1^)	Moisture content (%)	Active functional group
D113	Acrylic	0.45–1.25 ≧ 95	10.5	45–52	carboxyl
D150	Acrylic	0.315–1.25 ≧ 90	10	42–50	carboxyl
D152	Acrylic	0.315–1.25 ≧ 95	8	60–80	carboxyl
D155	Acrylic	0.315–1.25 ≧ 95	8.3	50–60	carboxyl

**TABLE 2:** Parameters of anion exchange rezsins.

Resin	Matrix	Particle size (mm) (%)	Exchange capacity (mmol g^−1^)	Moisture content (%)	Active functional group
D311	Acrylic	0.315–1.25 ≧ 95	6.5	48–58	amino
D314	Acrylic	0.315–1.25 ≧ 95	5.5	60–65	amino
D315	Acrylic	0.315–1.25 ≧ 95	7.2	58–65	amino
D318	Acrylic	0.45–1.25 ≧ 95	7	54–64	amino

### 2.2 MF and UF treatment of fermentation broth

MF membranes with a pore size of 0.1 μm were used to remove suspended solids, microorganisms, colloidal particles, and media based on a sieving mechanism, and UF membranes removed residual proteins, polysaccharides, pigments, and large molecular impurities from microfiltrates. Briefly, 2 L of fermentation broth was subjected to a full filtration cycle under intermittent crossflow to evaluate the effects of the membrane operating pressure, fermentation broth feed flow rate, and membrane module operating temperature on the separation efficiency of MF and UF. The optimal operating parameters were determined based on the average flux (Eqs 1, 2) and the flux recovery rate (Eq. 6), cell removal rate of the MF and UF membranes measured by a spectrophotometer (Eq. 3), removal rates pf protein (Eq. 4), and pigment (Eq. 5). The protein concentration was determined using the Coomassie Brilliant Blue method.

$$J_{V}=\frac{V}{ST}$$ (1)

$$\mathrm{Average flux}=\frac{V_{p}}{S∙T}$$ (2)

$$\mathrm{Cell removal}\left(\%\right)=\left(1-\frac{A_{2}}{A_{1}}\right)\times 100$$ (3)

$$\mathrm{Protein removal}\left(\%\right)=\left(1-\frac{A_{4}}{A_{3}}\right)\times 100$$ (4)

$$\text{Pigment }\text{removal}\left(\%\right)=\left(1-\frac{A_{6}}{A_{5}}\right)\times 100$$ (5)

$$\text{Recovery }\text{ratio}\left(\%\right)=\left(\frac{J_{v1}}{J_{v2}}\right)\times 100$$ (6)

where *J*_*V*_ is the membrane flux [L· (m^2^·h)⁻^1^], *V* is the filtrate volume (L), *S* is the effective membrane area (m^2^), *T* is the time (h), *V*_*p*_ is the permeate volume (L), *A*_*1*_ and *A*_*2*_ are the absorbance of the feed and permeate at 600 nm, respectively, *A*_*3*_ and *A*_*4*_ are the absorbance of the feed and permeate at 595 nm, respectively, *A*_*5*_ and *A*_*6*_ are the absorbance of the feed and permeate at 430 nm, respectively, *J*_*V1*_ is the pure water membrane flux after chemical cleaning [L/(m^2^·h)], and *J*_*V2*_ is net pure water membrane flux [L/(m^2^·h)].

### 2.3 Ion exchange resin purification of nylon 54 salt fermentation broth

#### 2.3.1 Resin selection

Before use, the ion exchange resins were soaked in ethanol and then the cationic resin was washed sequentially with 1.0 M hydrochloric acid, 1.0 M sodium hydroxide, and 1.0 M hydrochloric acid. The anion exchange resin was sequentially washed with 1.0 M sodium hydroxide, 1.0 M hydrochloric acid, and 1.0 M sodium hydroxide. The resins were then washed with deionized water until the washing solution was neutral. Then, 10 g of wet resin and 30 mL of 200 g/L pH-neutral nylon 54 salt fermentation broth were added to a 100 mL conical flask. The flask was then placed in a constant temperature shaker at 20°C and 200 rpm for 4 h to reach adsorption equilibrium. After adsorption, the adsorption liquid was filtered, and the filtrate concentration was determined using high-performance liquid chromatography (HPLC). The resins were then washed three times with deionized water. The cation exchange resins were desorbed with 25 mL of 80 g/L succinic acid solution, and the anion exchange resins were desorbed with 25 mL of 102 g/L (1.0 M) cadaverine solution. Organic acid succinic acid and organic amine cadaverine can efficiently exchange with resins adsorbing cadaverine cations and succinic acid anions, thereby synchronously producing the target product cadaverine succinate. This approach circumvents the use of acidic or alkaline solutions, minimizes waste salt production, and facilitates resin regeneration in subsequent steps. The flask was kept in a constant temperature shaker at 20°C for 4 h. The desorption solution was analyzed using HPLC. The resins were screened based on their equilibrium adsorption capacity (*Q*_*e*_, mg/g, Eq. 7), desorption capacity (*Q*_*d*_, mg/g, Eq. 8), and desorption rate (*D*, %, Eq. 9).

$$Q_{e}=\frac{\left(c_{0}-c_{e}\right)V_{i}}{W}$$ (7)

$$Q_{d}=\frac{c_{d}V_{d}}{W}$$ (8)

$$D=\frac{Q_{d}}{Q_{e}}\times 100\%$$ (9)

where *c*_*0*_ and *c*_*e*_ are the initial and equilibrium concentrations of the adsorption solution (mg/L), respectively, *V*_*i*_ is the total volume of the adsorption solution (L), *W* is the mass of the wet resin (g), *c*_*d*_ is the concentration of the desorption solution (mg/L), and *V*_*d*_ is the volume of the desorption solution (L).

#### 2.3.2 Adsorption isotherm determination

Three common isotherm models (Hoque et al., 2024), i.e., Langmuir (Eq. 10), Freundlich (Eq. 11), and Temkin-Pyzhev (Eq. 12) models, were used to evaluate the thermodynamic properties of resin adsorption of the nylon 54 salt. Static adsorption and isotherm simulation were performed at 20°C.

$$q_{e}=\frac{q_{m}K_{L}c_{e}}{1+K_{L}c_{e}}$$ (10)

$$q_{e}=K_{F}C_{e}^{1/n}$$ (11)

$$q_{e}=\frac{RT}{b_{T}}\ln\;\left(a^{T}C_{e}\right)$$ (12)

where *q*_*e*_ is the equilibrium adsorption capacity (mg/g), *q*_*m*_ is the maximum adsorption capacity (mg/g), *K*_*L*_ is the equilibrium adsorption constant (L/mg), *C*_*e*_ is the equilibrium adsorption concentration (mg/L), *K*_*F*_ and n are characteristic parameters of the equation, *a*_*T*_ is a thermodynamic constant (L/mg), and *b*_*T*_ is a thermodynamic constant (J/mol).

#### 2.3.3 Adsorption thermodynamics

The adsorption thermodynamics was studied to understand the adsorption mechanisms of the D150 and D315 resins (Mendel et al., 2023). Thermodynamic parameters, such as Gibbs free energy change (*ΔG*), enthalpy change (*ΔH*), and entropy change (*ΔS*), were calculated using the Clapeyron-Clausius relations (Eqs 13–15) (Wang et al., 2019).

$$\ln C_{e}=\frac{∆H}{RT}+C_{k}$$ (13)

∆G=−nRT (14)

$$∆S=\frac{∆H-∆G}{T}$$ (15)

where *C*_*k*_ is a constant, *R* is the universal gas constant (8.314 J/mol), *T* is the thermodynamic temperature (K), and *n* is the Freundlich coefficient.

#### 2.3.4 Adsorption kinetics

Adsorption efficiency is quantitatively described by adsorption kinetics, which plays an indispensable role in understanding adsorption mechanisms and designing adsorption equipment (Peng et al., 2023). The static adsorption data of the D150 and D315 resins were fitted by the pseudo-first-order kinetic model (Eq. 16) and pseudo-second-order kinetic model (Eq. 17) to obtain the adsorption kinetic models.

$$q_{t}=q_{e}\left(1-e^{-K_{1}t}\right)$$ (16)

$$q_{t}=\frac{K_{2}q_{e}^{2}t}{1+K_{2}q_{e}t}$$ (17)

where *q*_*t*_ is the amount of resin adsorbed at time t (mg/g), *t* is the instantaneous adsorption time (min), *K*_*1*_ is the pseudo-first-order rate constant [g/(mg·min)], and *K*_*2*_ is the pseudo-second-order rate constant [g/(mg·min)].

#### 2.3.5 Dynamic adsorption and desorption experiments

The pretreated resin was loaded onto a glass column with a volume of 30 mL. The inner diameter of the column was 2.0 cm and the heights were 14.0 cm. The flow rate was controlled using a peristaltic pump, and the effluent from the resin was collected quantitatively using a fraction collector (Ahmad et al., 2021). The nylon 54 salt fermentation liquid was passed through the glass column at different flow rates until reaching adsorption equilibrium. The D150 resin column was eluted with an aqueous succinic acid solution (80 g/L) at different flow rates, whereas the D315 resin column was eluted with an aqueous cadaverine solution (1 M) at different flow rates. The concentrations of the different volume fractions of the effluent were measured using HPLC. The breakthrough curves of cadaverine and succinic acid on the resin columns were plotted with the effluent column bed volume (BV, mL) on the *x*-axis and the concentrations of cadaverine and succinic acid in the effluent on the *y*-axis. The effects of nylon 54 salt concentration and flow rate on the dynamic breakthrough curves were investigated. In the dynamic adsorption experiment, the amounts of cadaverine and succinic acid adsorbed per unit resin were determined by integrating the area above the breakthrough curve (Eqs 18, 19).

$$m_{a}=\int_{0}^{V_{t}}\left(C_{0}-C_{ta}\right)dV$$ (18)

$$Q_{a}=\frac{m_{a}}{m_{s}}$$ (19)

where *m*_*a*_ is the total mass of cadaverine or succinic acid adsorbed by the resin column (g), *C*_*ta*_ is the concentration of cadaverine or succinic acid in the effluent at a given time (g/L), *V*_*t*_ is the volume of the effluent corresponding to *C*_*ta*_ (mL), *Q*_*a*_ is the amount of cadaverine or succinic acid adsorbed per unit of resin (mg/g), and *m*_*s*_ is the amount of resin used (g).

### 2.4 Crystallization of nylon 54 salt

The nylon 54 salt simulated solution and the actual solution eluted from the ion exchange resin were concentrated and placed into jacketed beakers, heated to 50°C, then cooled to 0°C. Then, ethanol was added during cooling at a rate of 1 mL/min with an addition amount six times the volume of the concentrate. Nylon 54 crystals were observed after precipitating for 1 h (Law et al., 2019). After the crystallization was complete, the product was filtered, washed, and dried. The experiment was repeated thrice under the same operating conditions to determine the average purity and yield of the product. The purity and yield were calculated using Eqs 20, 21, respectively.

$$Purity=\frac{m_{1}}{M_{1}}\times 100\%$$ (20)

$$Yield=\frac{m_{1}}{M_{0}}\times 100\%$$ (21)

where *m*_*1*_ is the actual measured mass of nylon 54 in the final product (g), *M*_*1*_ is the total mass of the final product (g), and *M*_*0*_ is the mass of nylon 54 in the initial solution (g).

### 2.5 HPLC conditions

A YMC carotenoid column (250 mm × 4.6 mm, s-5 μm, Grace, Columbia, MD, United States) was used to detect cadaverine on a differential detector (1260, Agilent Technologies, Santa Clara, CA, United States) at 35°C. The mobile phase consisted of a mixture of 5% acetonitrile and 0.5% trifluoroacetic acid. The flow rate was 0.6 mL min^−1^, and the injection volume was 10 μL.

An HPX-87H ion-exclusion column (300 mm × 7.8 mm) was used to detect succinic acid on the differential detector at 35°C. The mobile phase contained 0.008 M H_2_SO_4_. The flow rate was 0.6 mL/min, and the injection volume was 20 μL.

## 3 Results and discussion

### 3.1 Microfiltration treatment of fermentation broth

A parametric study was conducted to optimize the membrane performance during MF treatment of fermentation broth. The effects of different parameters, including transmembrane pressure (TMP), feed flow rate, and feed temperature, were examined to determine the optimal operating parameters.

#### 3.1.1 Effect of TMP

**FIGURE 2:**
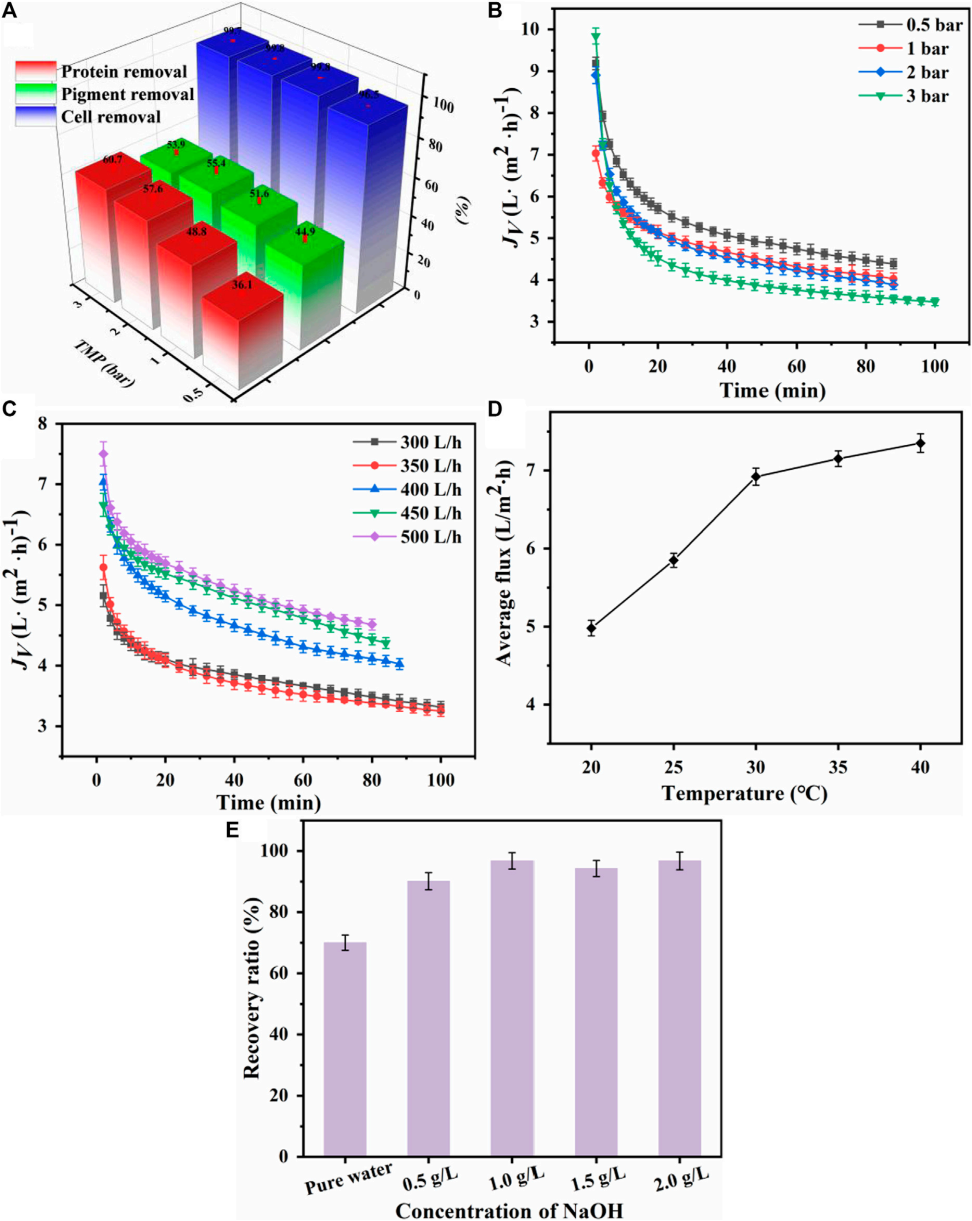
Effects of transmembrane pressure (TMP) on **(A)** microfiltration performance and **(B)** membrane flux. Effect of feed flow rate on **(C)** microfiltration membrane flux. Effect of feed temperature on **(D)** microfiltration membrane flux. Effect of NaOH concentration on **(E)** the recovery of microfiltration membrane flux.

In the process of membrane separation of cells and proteins from the fermentation broth, Saha et al. (2017) and Miao et al. (2020) reported that as the TMP increased, the permeate flux increased. However, when the operating pressure exceeds the critical pressure, the permeate flux does not increase, indicating the presence of an optimal pressure for achieving the maximum permeate flux. Therefore, four different TMPs were selected for MF membranes. As shown in Figure 2A, as TMP increased, the removal efficiency of proteins and pigments significantly increased because the macromolecules deposited on the membrane and formed a cake layer. As shown in Figure 2B, the initial flux increased with increasing TMP. A higher TMP can provide a greater driving force for solvent permeation, but it can also cause macromolecules to deposit on the membrane or clog the membrane pores, thereby increasing membrane fouling and reducing flux. Therefore, considering the energy consumption and the membrane fouling, a TMP of 1 bar was selected for the MF treatment.

#### 3.1.2 Effect of feed flow rate

The membrane performance also depends on the feed flow rate. Loulergue et al. (2018). Reported that as the feed flow rate increased, the Reynolds number increased, which decreased the boundary layer thickness and the corresponding thermal polarization and concentration polarization, thus reducing the resistance. As shown in Figure 2C, when the MF feed flow rate was increased from 300 to 450 L/h, the flux increased significantly by 50%; however, no significant changes were observed when the flow rate was further increased to 500 L/h. Therefore, a feed flow rate of 450 L/h was selected for MF.

#### 3.1.3 Effect of feed temperature

Previous studies (Santoro et al., 2017) reported that the feed temperature was one of the most important parameters affecting membrane performance. As shown in Figure 2D, the average MF flux increased with the feed temperature because of the decreased viscosity of the nylon 54 salt fermentation broth (Wen et al., 2023). The high fluidity of the fermentation broth at an elevated temperature improved the flux during the membrane filtration process. Controlling the MF feed temperature at 30°C could achieve a high membrane filtration flux.

#### 3.1.4 Membrane fouling

A continuous decline in membrane flux was observed in the MF experiments of the fermentation broth. The reduction in flux was primarily due to the biofouling caused by microorganisms, proteins, and colloidal particles. These solid particles accumulated on the membrane surface and formed a biomass cake, significantly increasing membrane resistance. As reported by Li et al. (2020), membrane flux could not be restored by simple pure water cleaning. The recovery of the MF membrane flux could reach 70% after cleaning with pressurized pure water and 93.5% after cleaning with a sodium hydroxide solution under pressure. Therefore, chemical cleaning is more effective than pure water cleaning. Different concentrations of sodium hydroxide solutions were used to clean the membrane under a TMP of 2 bar. As shown in Figure 2E, the best recovery (96.76%) was achieved with a 1 g/L NaOH solution.

### 3.2 Determining UF conditions

#### 3.2.1 Effect of TMP

**FIGURE 3:**
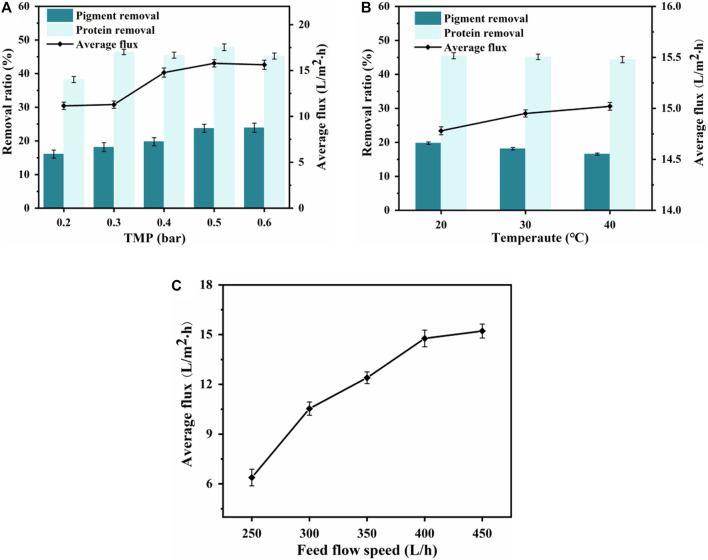
Effect of transmembrane pressure (TMP) on **(A)** ultrafiltration performance. Effect of feed temperature on **(B)** ultrafiltration performance. Effect of feed flow rate on **(C)** ultrafiltration membrane flux.

To obtain the optimal pressure for maximum permeate flux (Miao et al., 2020), five different TMPs were selected to study the effect of TMP on the separation efficiency of the nylon 54 salt. As shown in Figure 3A, when the filtrate was in the mass-transfer control zone, increasing the TMP enhanced the driving force for filtration and significantly improved the UF flux. However, as the TMP increased, a gel layer formed on the membrane surface and gradually became compressed and thicker, thereby increasing the resistance and causing the flux to increase slowly and then decrease. Therefore, a TMP of 0.4 bar was chosen for the UF separation.

#### 3.2.2 Effect of feed flow rate

The effects of different feed flow rates on UF flux were investigated, and the average fluxes are shown in Figure 3C. As the feed flow rate increased, the concentration polarization effect was significantly reduced, increasing the flux (Loulergue et al., 2018). When the feed flow rate was 400 L/h, the effect on UF flux was minimal. Therefore, a feed flow rate of 400 L/h was selected.

#### 3.2.3 Effect of feed temperature

The effects of different feed temperatures in the 20°C–40°C range on the permeation flux of the UF membrane and the removal rates of protein and pigment by the UF membrane are shown in Figure 3B. Because the previous MF removed most of the suspended particles, the average UF flux gradually increased with temperature; however, the protein and pigment removal rates both showed a downward trend. Therefore, the UF feed temperature was controlled at 30°C to achieve a balance of flux and removal rate (Santoro et al., 2017).

### 3.3 Decolorization treatment

Activated carbon treatment is a common technique to remove impurities and pigments that cause the dark color of the fermentation broth (Lee S. Y. et al., 2019). Three different types of activated carbon were tested in this study. The decolorization temperature and time were optimized to achieve a high decolorization efficiency relative to the amount of activated carbon added. The color removal was evaluated by measuring the optical density of the solution at 430 nm. Powdered lignin-based activated carbon was selected in this study, as it could achieve almost complete color removal (Supplementary Figure S1). Approximately 1.5% (w/v) powdered lignin-based activated carbon was sufficient to completely decolorize the fermentation broth (Supplementary Figure S2). The high efficiency was due to the low density and high specific surface area of the lignin-based activated carbon, which offered excellent adsorption performance and low resistance loss. The optimal decolorization temperature and time were 40°C (Supplementary Figure S3) and 50 min (Supplementary Figure S4), respectively.

Following MF, UF, and decolorization, the nylon 54 salt fermentation broth exhibited a sterilization rate of ≥99.83%, a protein concentration of ≤40 mg·L^−1^, and an absorbance of ≤0.021 at 430 nm. The recovery rates of cadaverine and succinic acid during pretreatment were 97%. The photographs for each operation during the pretreatment process are shown in Supplementary Figure S5.

### 3.4 Simultaneous purification of nylon 54 salt solution by ion exchange resin

#### 3.4.1 Screening of ion exchange resins

Adsorption and desorption capacities and desorption ratios are the most critical indicators for evaluating the recovery of the target substances. The optimal macroporous ion exchange resin was identified by investigating the adsorption and desorption capacities and desorption ratios.

**FIGURE 4:**
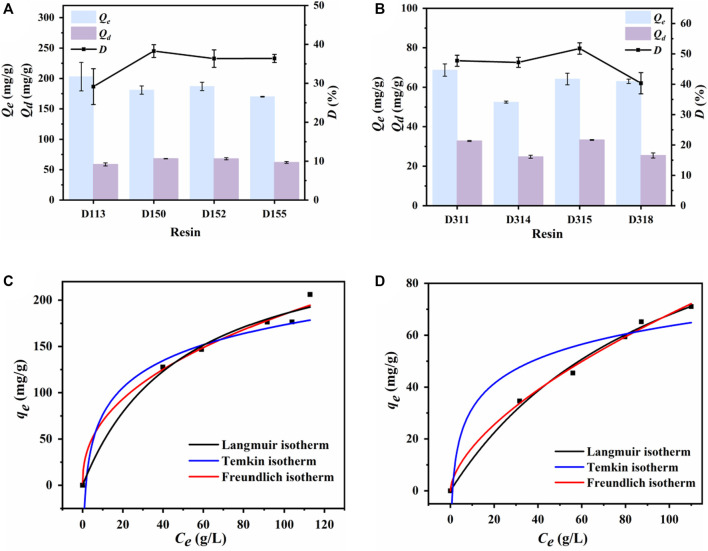
Adsorption and desorption capacity of different **(A)** cation exchange resins and **(B)** anion exchange resins. Langmuir, Freundlich, and Temkin-Pyzhev adsorption isothermal curves of **(C)** D150 cation exchange resin for cadaverine and **(D)** D315 anion exchange resin for succinic acid.

The cation-resin screening results are shown in Figure 4A. D113 exhibited the highest cadaverine adsorption capacity, which could be attributed to its highest exchange capacity (Table 1). However, D150 exhibited the highest desorption capacity and desorption ratio, with its exchange capacity second only to that of D113. Therefore, D150 was selected for further analysis.

The anion–resin screening results are shown in Figure 4B. D311 exhibited the highest succinic acid adsorption capacity. However, D315 exhibited the highest desorption capacity and ratio, which could be attributed to its highest exchange capacity (Table 2) (Din et al., 2021). Therefore, D315 was selected for further analysis.

#### 3.4.2 Adsorption isotherm

Three common isotherm models were used to examine the thermodynamic properties of nylon 54 salt adsorption by the D150 and D315 resins. Static adsorption and isotherm simulation were conducted at 20°C.

The results for the cationic resin D150 are shown in Figure 4C; Supplementary Table S1. Compared with the Temkin-Pyzhev adsorption isotherm, the correlation coefficients (R^2^) obtained from Langmuir and Freundlich equations were higher than 0.98, and the Freundlich equation had the highest R^2^, indicating a best fitting. Therefore, the adsorption of cadaverine by the D150 resin was a multilayer adsorption process. The Freundlich index (n) represents the feasibility of adsorption, where n > 1 indicates good adsorption. In most cases, n values between 1 and 10.34 represent positive absorption. In this study, the n value varied between 1.99 and 2.71, demonstrating good cadaverine adsorption on the resin (Ding et al., 2011). The q_m_ value predicted using the Langmuir model (199.73 mg/g) also confirmed a good fit.

The results for the anion resin D315 are shown in Figure 4D; Supplementary Table S1. The Langmuir and Freundlich equations showed relatively good fits (R^2^ > 0.98), but the Freundlich equation had the highest R^2^, indicating that the adsorption of succinic acid by the D315 resin was also a multilayer adsorption process. The n value of D315 varied between 1.52 and 1.76, demonstrating good adsorption performance of succinic acid on the resin (Ding et al., 2011). The q_m_ predicted by the Langmuir model (100.46 mg/g) also confirmed a good fit.

#### 3.4.3 Adsorption thermodynamics

The adsorption thermodynamics was studied to analyze the adsorption mechanisms of the D150 and D315 resins and determine whether the adsorption process can occur spontaneously (Phojaroen et al., 2024). Due to the strong correlation with the Freundlich equation, the linear fit based on the Clapeyron-Clausius formula is presented (Supplementary Figures S6, S7). The thermodynamic parameters at 20°C are listed in Table 3.

**TABLE 3:** Thermodynamic parameters for the adsorption of cadaverine on D150 and succinic acid on D315 at 20°C.

Resin	ΔH(KJ/mol)	ΔS (J/mol)	ΔG (KJ/mol)	R^2^
D150	−1.40	14.76	−5.728	0.9913
D315	−1.71	7.80	−3.997	0.9888

The negative ΔG values indicate that the adsorption processes of cadaverine and succinic acid on the resins are thermodynamically favorable. Based on the negative ΔH values, the adsorptions of cadaverine and succinic acid are supposed to be exothermic, and lower temperatures favor adsorption, which is consistent with the experiments. The positive ΔS values suggest that the disorder at the solid-liquid interface increases after adsorption, and the cadaverine and succinic acid adsorbed on the resin surface are arranged more randomly after adsorption (Bilgili, 2006).

#### 3.4.4 Adsorption kinetics

**FIGURE 5:**
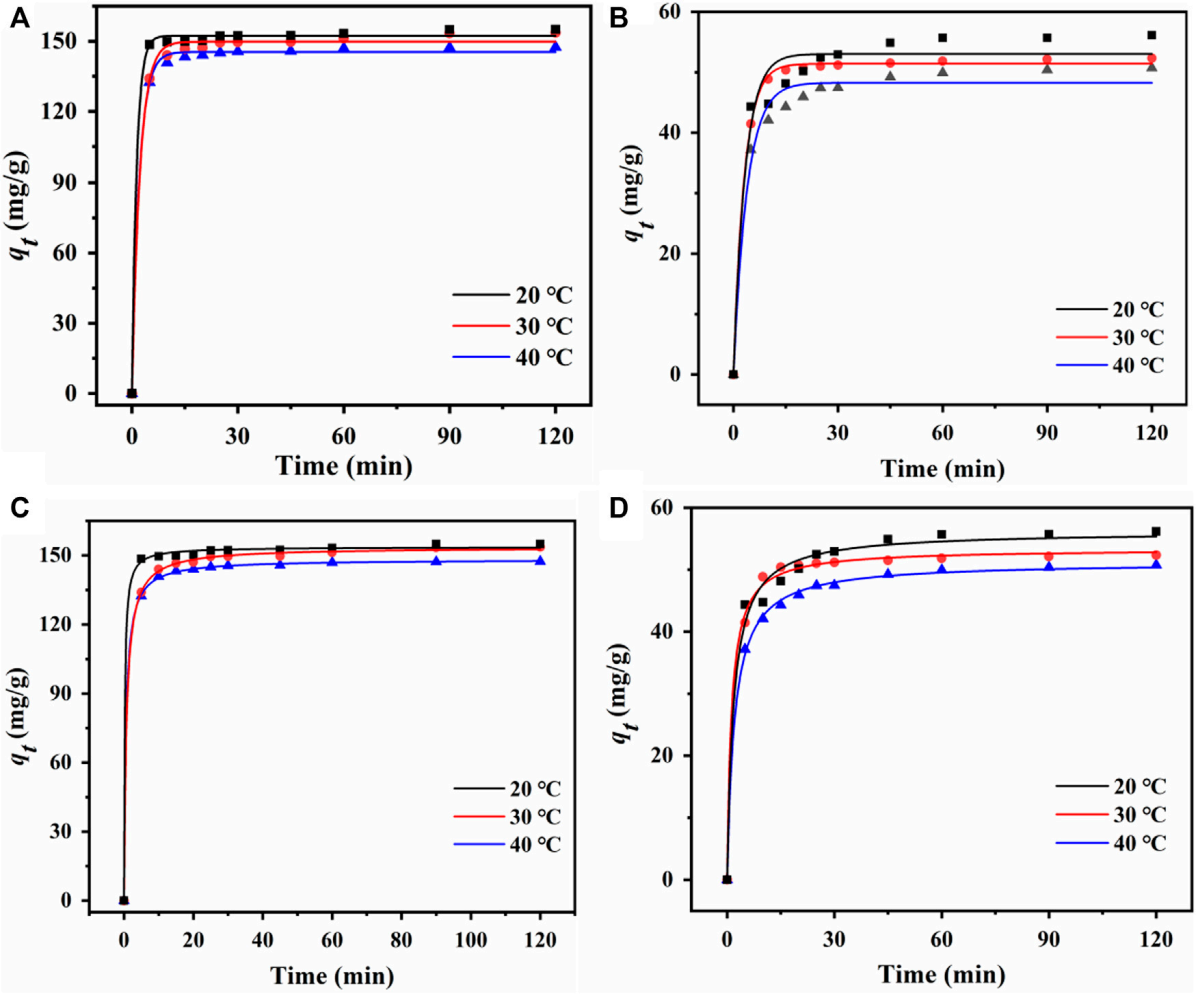
Pseudo-first-order kinetic curves of **(A)** D150 and **(B)** D315 at different temperatures. Pseudo-second-order kinetic curves of **(C)** D150 and **(D)** D315 at different temperatures.

Adsorption efficiency can be quantitatively described by adsorption kinetics. The cadaverine adsorption data and fittings using two kinetic models are shown in Figures 5A, C; Supplementary Table S2. The pseudo-second-order kinetic model could fit the cadaverine adsorption data better than the pseudo-first-order kinetic model with higher R^2^ values at different temperatures. The equilibrium adsorption calculated using the pseudo-second-order kinetic model could describe the adsorption of cadaverine on the resin more accurately, indicating that the adsorption was mainly chemisorption, which is consistent with the characteristics of ion exchange adsorption (Peng et al., 2023).

The succinic acid adsorption data and fittings using two kinetic models are shown in Figures 5B, D; Supplementary Table S3. Similarly, the pseudo-second-order kinetic model fits the adsorption kinetics data better than the pseudo-first-order kinetic model, indicating the adsorption of succinic acid on the resin was also dominated by chemisorption (Peng et al., 2023).

### 3.5 Dynamic adsorption and desorption

#### 3.5.1 Optimization of dynamic adsorption process

Fixed-bed columns are commonly used in continuous industrial chromatographic separation processes to achieve real-time control and improve efficiency. The performance of the fixed-bed column adsorption system was evaluated by a dynamic adsorption study. The breakthrough point was used to determine the optimal feed flow rate and concentration for dynamic adsorption (Zhou et al., 2023). The dynamic breakthrough curves for D150 and D315 were constructed using a 158 g/L nylon 54 salt solution.

As shown in Supplementary Figure S8, the maximum adsorption capacity (194.49 mg/g dry resin) was observed for the D150 column at the lowest flow rate (2 BV/h) with a breakthrough point of 3.2 BV. The optimal adsorption performance at 2 BV/h required a breakthrough time of 1.6 h, approximately 1.3 times and 1.6 times longer than those at flow rates of 3 and 4 BV/h, respectively. As the D150 column had a relatively high cadaverine absorption at 3 BV/h (194.14 mg/g dry resin) and a reasonable operation time (1.2 h), the optimal flow rate of 3 BV/h was chosen, with a breakthrough point of 3.6 BV (Zhuang et al., 2020).

As shown in Supplementary Figure S9, the maximum adsorption capacity was 100.46 mg/g dry resin for the D315 column at the slowest flow rate (2 BV/h) with a breakthrough point of 3 BV. The optimal adsorption performance at 2 BV/h required a breakthrough time of 1.5 h, approximately 1.3 times and 1.6 times longer than those at feed flow rates of 3 and 4 BV/h, respectively. Because the D315 column had relatively high succinic acid absorption at 3 BV/h (99.36 mg/g dry resin) and a reasonable operation time (1.1 h), an optimal flow rate of 3 BV/h was chosen, with a breakthrough point of 3.4 BV. Considering the cation and anion columns, the flow rate was fixed at 3 BV/h for the simultaneous separation of cadaverine and succinic acid (Zhuang et al., 2020).

**FIGURE 6:**
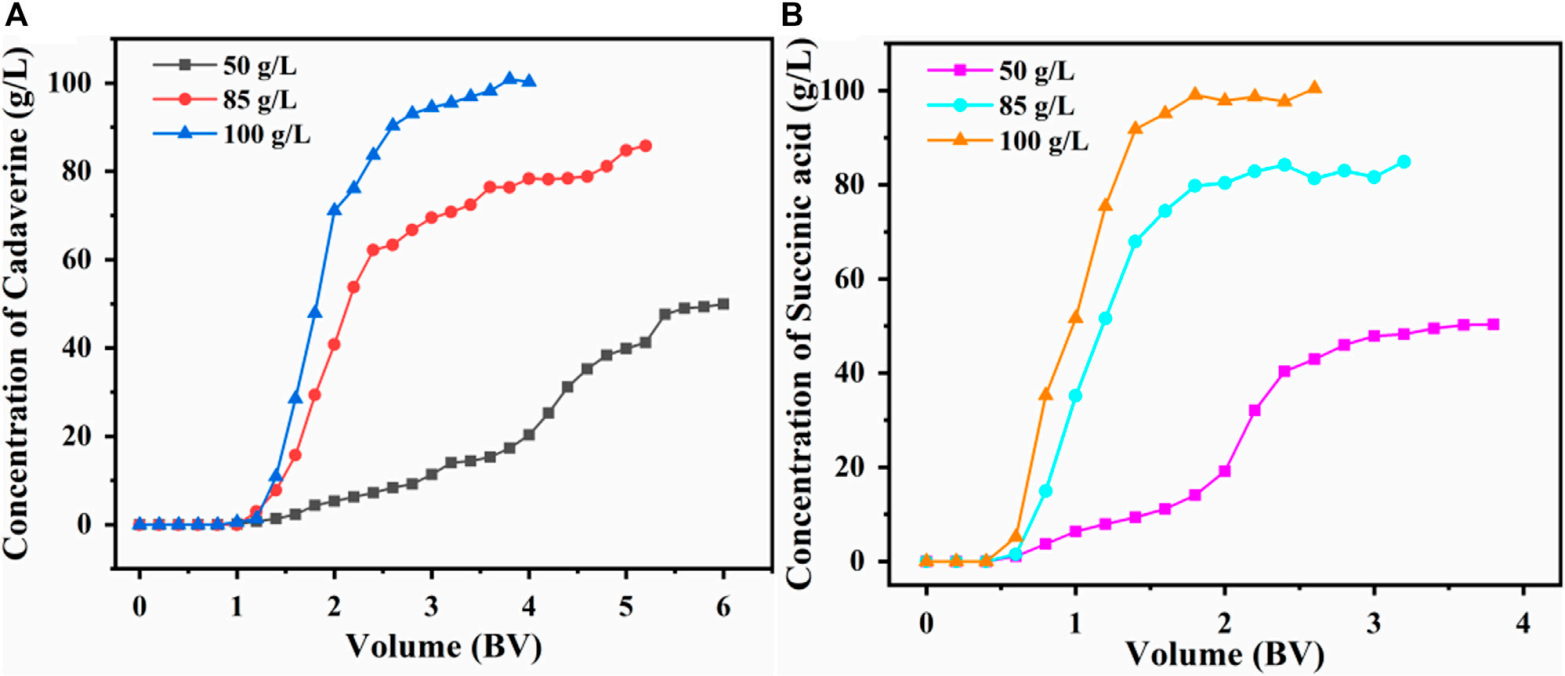
Effect of **(A)** initial cadaverine concentration and **(B)** succinic acid concentration on dynamic adsorption.

As the initial concentrations of cadaverine and succinic acid increased, the permeation decreased as shown in Figure 6. This phenomenon was attributed to the slow mass-transfer process and the delayed breakthrough time at low concentrations (Sumona et al., 2022). With the increasing cadaverine and succinic acid concentrations, the mass-transfer interface decreased, and the slope of the permeation curve increased with leftward deviation.

As shown in Figure 6A, the final adsorption capacities of the D150 column were 196.56, 199.73, and 194.14 mg/g for the initial cadaverine concentrations of 50, 85, and 100 g/L, respectively. The adsorption capacity was the highest at a cadaverine concentration of 85 g/L; therefore, 85 g/L was chosen as the optimal cadaverine concentration for the D150 column.

As shown in Figure 6B, the final adsorption capacities of the D315 column were similar for different succinic acid concentrations (100.22, 99.36, and 101.64 mg/g for 50, 85, and 100 g/L, respectively). Considering that the amount of feed solution required for a higher concentration (100 g/L) was lower, and the time required for a complete breakthrough was shorter, a succinic acid concentration of 100 g/L was selected as the optimal fermentation broth for the D315 column.

In summary, the optimal fermentation broth loading concentration was determined to be a nylon 54 salt concentration of 183 g/L for simultaneous separation on a chromatographic column.

#### 3.5.2 Optimization of the dynamic desorption process

**FIGURE 7:**
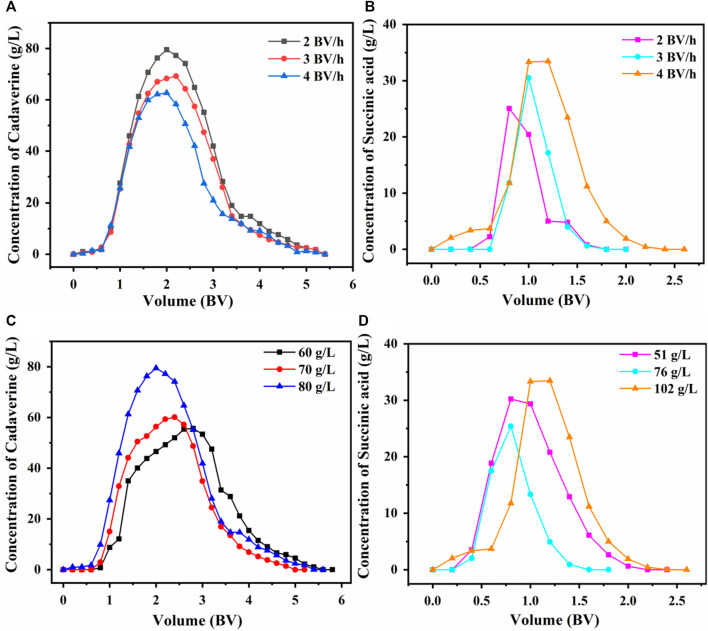
Effect of flow rate on dynamic desorption of **(A)** D150 and **(B)** D315. Effects of **(C)** succinic acid concentration on the dynamic desorption of D150 and **(D)** cadaverine concentration on the dynamic desorption of D315.

The desorption results of using the D150 column with 80 g/L succinic acid at flow rates of 2, 3, or 4 BV/h are shown in Figure 7A. The maximum desorption concentration of cadaverine at 2 BV/h was significantly higher than those at 3 and 4 BV/h (Du et al., 2012). The cadaverine concentration decreased with an increase in column flow rate because a low flow rate allowed cadaverine to diffuse in and out of the pores of the ion exchanger as they bind to or dissociate from the column (Khodwe et al., 2022). Therefore, 2 BV/h was selected as the optimal desorption flow rate to achieve a higher desorption concentration. The volume required for complete desorption of cadaverine was 5 BV.

The desorption results of using the D315 column with 102 g/L (1 M) of succinic acid at flow rates of 2, 3, or 4 BV/h are shown in Figure 7B. At 4 BV/h, the maximum desorption concentration of succinic acid was significantly higher than those at 2 and 3 BV/h. Therefore, 4 BV/h was selected as the optimal desorption flow rate for achieving a sharp peak within a short time. The volume required for complete desorption of succinic acid was 2.5 BV.

At a desorption flow rate of 2 BV/h, the cadaverine in the D150 column was desorbed using 60, 70, or 80 g/L of succinic acid. As shown in Figure 7C, when the succinic acid concentration was 80 g/L, the maximum cadaverine concentration in the eluent was 79.5 g/L (Ding et al., 2024). Therefore, 80 g/L succinic acid was chosen as the optimal desorption concentration for the D150 resin.

At a desorption flow rate of 4 BV/h, the succinic acid in the D315 column was desorbed using 51 g/L (0.5 M), 76 g/L (0.75 M), and 102 g/L (1 M) cadaverine solutions. As presented in Figure 7D, at a cadaverine concentration of 102 g/L (1 M), the maximum succinic acid concentration in the eluent was 33.46 g/L (Ding et al., 2024). Therefore, 102 g/L (1 M) was chosen as the optimal cadaverine concentration for the desorption of D315 resin.

#### 3.5.3 Simultaneous purification of real nylon 54 salt fermentation broth

**FIGURE 8:**
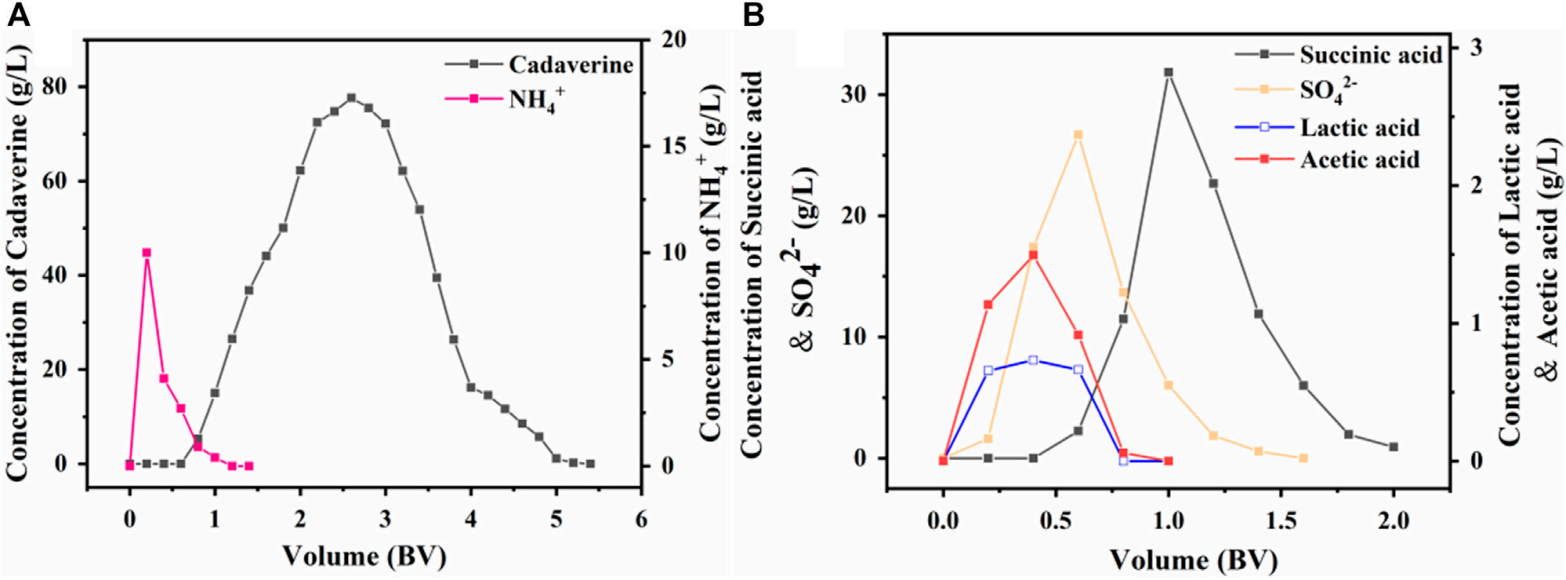
Dynamic desorption performance of **(A)** D150 and **(B)** D315.

A real fermentation broth with a nylon 54 salt concentration of 183 g/L was applied sequentially through a D150 cation exchange column and a D315 anion exchange column using an optimal sample flow rate of 3 BV/h and a sample volume of 3.6 BV. The cation exchange column was eluted with an 80 g/L succinic acid aqueous solution at an optimal flow rate of 2 BV/h and an eluent volume of 5 BV. As shown in Figure 8A, the NH_4_^+^ removal rate reached 98.90%, and the cadaverine recovery rate was 98.01% when the recovery volume segment was in the 1.2–4.8 BV range.

The anion exchange column was eluted with a 102 g/L (1 M) cadaverine solution at an optimal flow rate of 4 BV/h and an eluent volume of 2.5 BV. Each elution was performed to remove as many impurities as possible from the fermentation solution, and the impurities were monitored in real time. The results in Figure 8B show that succinic acid had the strongest binding capacity and was eluted last with some overlapping peaks with SO_4_^2−^ (Wu et al., 2021). Therefore, when the recovery volume segment was in the 1–1.8 BV range, acetic acid and lactic acid could be completely removed; the SO_4_^2−^ removal rate reached 77.79%, and the succinic acid recovery rate was 89.35%.

### 3.6 Nylon 54 salt crystallization

**FIGURE 9:**
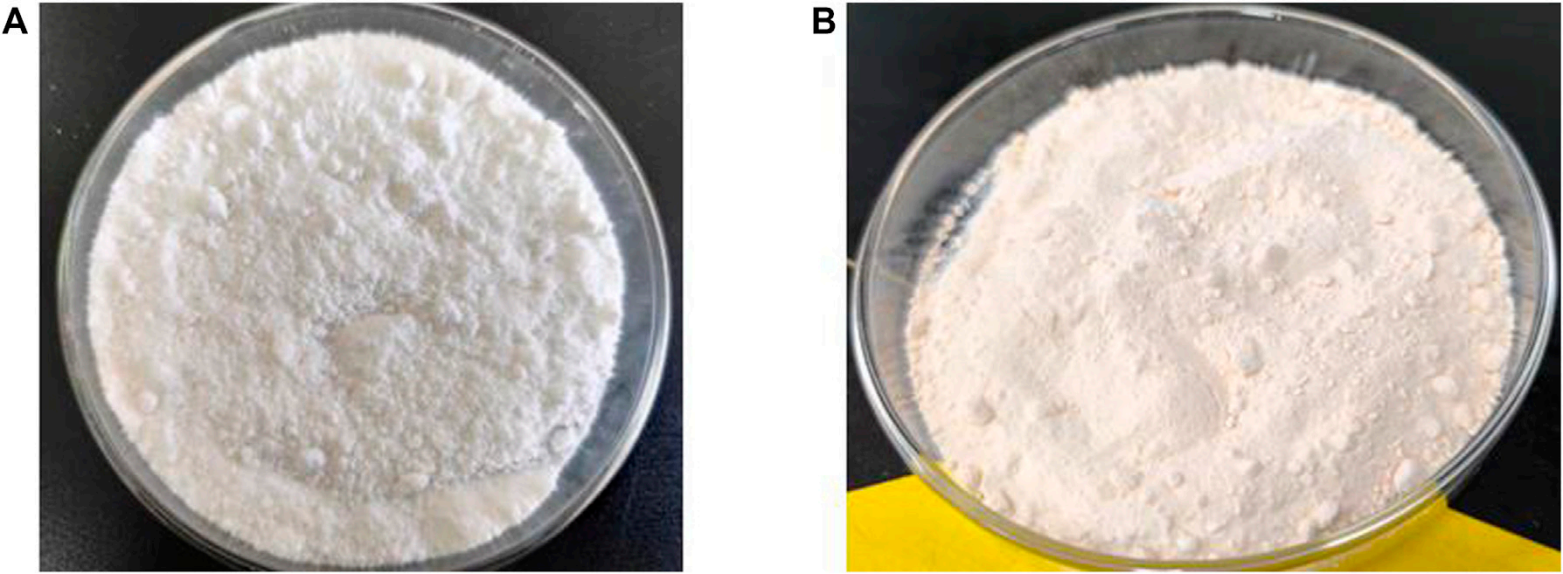
Appearance of purified nylon 54 salt product from **(A)** simulated fermentation broth and **(B)** real fermentation broth.

Crystallization experiments were conducted using simulated and actual fermentation broth elution liquids (2000 g/L). When the temperature was reduced to 0°C, the simulated solution achieved a nylon 54 yield of 58% with a purity of 99.16%, and the cadaverine to succinic acid molar ratio was 1.11:1. In an actual fermentation broth, nylon 54 was obtained with a yield of 56.21% and a purity of 98.10%, and the cadaverine to succinic acid molar ratio was 1.01:1. The appearance of the crystallized products is shown in Figure 9. The crystals derived from the simulated fermentation broth were white, whereas those from the actual fermentation broth exhibited a slightly yellowish tinge on their surfaces.

**FIGURE 10:**
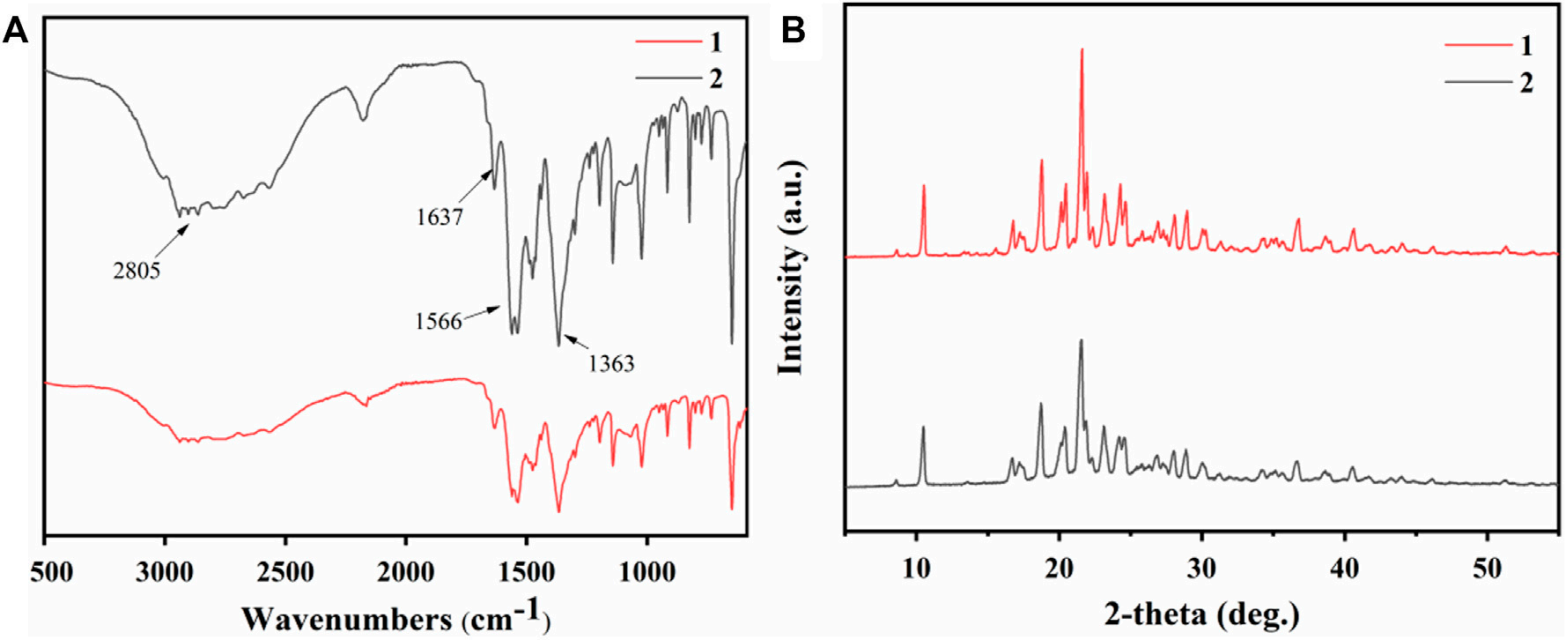
**(A)** Infrared spectra and **(B)** PXRD patterns of crystallization products from simulated fermentation broth (1) and real fermentation broth (2).

The crystallization products of the two raw materials were characterized by infrared spectroscopy. As shown in Figure 10A, the N-H stretching vibration absorption peak shifted significantly to lower frequencies after the reaction between amines and carboxylic acids, overlapping with the C-H stretching absorption peak and forming a broad and strong band in the 3,200–2,500 cm^−1^ range. The strong absorption peak in the 1,600–1,500 cm^−1^ range was related to the deformation vibration of the N-H group. The C-N and C=O groups showed stretching vibration peaks in the 1,230–1,050 cm^−1^ and 1,650–1,400 cm^−1^ ranges, respectively. The C-O stretching vibration was observed at 1,363 cm^−1^.

The products obtained by crystallization from different raw materials were characterized by powder X-ray diffraction (PXRD), as illustrated in Figure 10B. The primary diffraction peaks appeared consistently for both products, corresponding to their crystal structures. Notably, the nylon 54 obtained from simulated fermentation broth had sharper main peaks and reduced impurity peaks, suggesting its higher crystallinity (Yang et al., 2019).

## 4 Conclusion

An integrated process for the separation and extraction of nylon 54 salt was developed in this study to enable synchronous isolation of succinic acid and cadaverine from the fermentation broth. The operational parameters were optimized to maximize the separation efficiency. The pretreatment process included MF at 1 bar pressure with a feed flow rate of 450 L/h at 30°C and UF at 0.4 bar with a feed flow rate of 400 L/h at 30°C. Then, 1.5% (m/v) powdered wood-activated carbon was used for decolorization at 40°C for 50 min. Membrane fouling was effectively cleaned using a 1 g/L NaOH solution at a pressure of 2 bar, resulting in a membrane flux recovery rate of up to 97.3%. Among eight tested resins, D150 and D315 were selected for the separation of cadaverine and succinic acid, respectively. Using real fermentation broth with a nylon 54 salt concentration of 183 g/L, sample flow rate of 3 BV/h, and sample volume of 3.6 BV, the solution was passed through a D150 cation exchange resin followed by a D315 anion exchange resin. Cation exchange chromatography was performed with an 80 g/L succinic acid solution at an optimal flow rate of 2 BV/h and an eluent volume of 5 BV. Anion exchange chromatography was performed using a 1 M cadaverine solution at an optimal flow rate of 4 BV/h with an eluent volume of 2.5 BV. These resins could effectively remove impurities. The static adsorption process of the resin followed both adsorption thermodynamics and adsorption kinetics theoretical models. The integrated process yielded nylon 54 salts with purities of 99.16% and 98.10% from the simulated and real fermentation broths, respectively. This green and efficient simultaneous separation and extraction method for nylon 54 has the potential to be extended to other biosynthesized polymers.

## Data Availability

The original contributions presented in the study are included in the article/Supplementary Material, further inquiries can be directed to the corresponding authors.
