# Supplementary material for: Separation and purification of nylon 54 salts from fermentation broth by an integrated process involving microfiltration, ultrafiltration, and ion exchange
Source: Front Bioeng Biotechnol. 2024 Aug 1;12:1448927. doi: 10.3389/fbioe.2024.1448927 (PMC11324497; doi:10.3389/fbioe.2024.1448927)
Supplement: Supplementary file 1 [file DataSheet1.docx]

**Separation and purification of nylon 54 salts from fermentation broth by an integrated process involving microfiltration, ultrafiltration, and ion exchange**

Xiaojie Zhaoª, Qixu Huª, Yue Yang ª, Jiao Feng ª, Xin Wang ª, Ganlu Li^a^, Hui Li^a^, *, kequan Chen ^a^ *

a State Key Laboratory of Materials-Oriented Chemical Engineering, College of Biotechnology and Pharmaceutical Engineering, Nanjing Tech University, Nanjing 211816, Jiangsu, China

Corresponding Author, E-mail: lihuill@njtech.edu.cn; kqchen@njtech.edu.cn

**List of table captions**

Table S1. Relevant parameters of the Langmiur, Freundlich and Temkin-Pyzhev adsorption isothermal models.

Table S2. Relevant parameters of pseudo first-order and pseudo second-order models of D150 at different temperatures.

Table S3. Relevant parameters of pseudo first-order and pseudo second-order models of D315 at different temperatures.

**List of figure captions**

Figure S1. Effect of activated carbon types on decolorization.

Figure S2. Effect of activated carbon dosage(w/v) on decolorization.

Figure S3. Effect of temperature on decolorization.

Figure S4. Effect of time on decolorization.

Figure S5. Sample photos of each operation during the pretreatment process. (a) Nylon 54 salt fermentation broth. (b) Permeate for membrane separation (c) Filtrate for decolorization of activated carbon.

Figure S6. Linear fitting of the Clapeyron-Clausius equation for D150.

Figure S7. Linear fitting of the Clapeyron-Clausius equation for D315.

Figure S8.  Effect of flow rate on dynamic adsorption of D150.

Figure S9.  Effect of flow rate on dynamic adsorption of D315.

Table S1. Relevant parameters of the Langmiur, Freundlich and Temkin-Pyzhev adsorption isothermal models.

| Model | Parameters | D150 | D315 |
| --- | --- | --- | --- |
| Langmiur | K_L_(L·mg^-1^） | 50.68 | 103.22 |
|  | q_m_ | 279.06 | 137.93 |
|  | R^2^ | 0.9836 | 0.9930 |
| Freundlich | K_F_[(mg·g^-1^)·(mg·L^-1^)^1/n^] | 26.10 | 4.09 |
|  | n | 2.35 | 1.64 |
|  | R^2^ | 0.9873 | 0.9948 |
| Temkin-Pyzhev | K_T_(Lꞏmg^−1^) | 0.61 | 1.00 |
|  | b_T_(Jꞏmol^−1^) | 42.20 | 13.80 |
|  | R^2^ | 0.9287 | 0.8814 |

Table S2. Relevant parameters of pseudo first-order and pseudo second-order models of D150 at different temperatures.

| T (℃) | q_e,exp_  (mg⋅g^-1^) | Pseudo-1st-order | | | Pseudo-2nd-order | | |
| --- | --- | --- | --- | --- | --- | --- | --- |
|  |  | q_e,cal_ (mg.g^-1^) | K_1_  (min^-1^) | R^2^ | q_e,cal_  (mg.g^-1^) | K_2_  [g·(mg⋅min)^-1^] | R^2^ |
|  |  |  |  |  |  |  |  |
| 20 | 155.03 | 152.27 | 0.7367 | 0.9983 | 155.16 | 0.0298 | 0.9993 |
| 30 | 153.66 | 149.75 | 0.4383 | 0.9965 | 152.85 | 0.0090 | 0.9996 |
| 40 | 147.46 | 145.36 | 0.4710 | 0.9984 | 148.28 | 0.0117 | 0.9999 |

Table S3. Relevant parameters of pseudo first-order and pseudo second-order models of D315 at different temperatures.

| T (℃) | q_e,exp_  (mg⋅g^-1^) | Pseudo-1st-order | | | Pseudo-2nd-order | | |
| --- | --- | --- | --- | --- | --- | --- | --- |
|  |  | q_e,cal_ (mg.g^-1^) | K_1_  (min^-1^) | R^2^ | q_e,cal_  (mg.g^-1^) | K_2_  [g·(mg⋅min)^-1^] | R^2^ |
|  |  |  |  |  |  |  |  |
| 20 | 56.14 | 53.02 | 0.2969 | 0.9593 | 56.17 | 0.0100 | 0.9888 |
| 30 | 52.32 | 51.41 | 0.3224 | 0.9986 | 53.32 | 0.0153 | 0.9973 |
| 40 | 50.73 | 48.27 | 0.2567 | 0.9793 | 51.21 | 0.0095 | 0.9987 |





Figure S1. Effect of activated carbon types on decolorization.





Figure S2. Effect of activated carbon dosage(w/v) on decolorization.





Figure S3. Effect of temperature on decolorization.





Figure S4. Effect of time on decolorization.


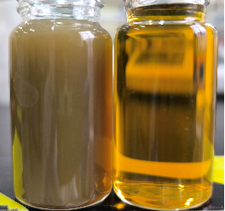

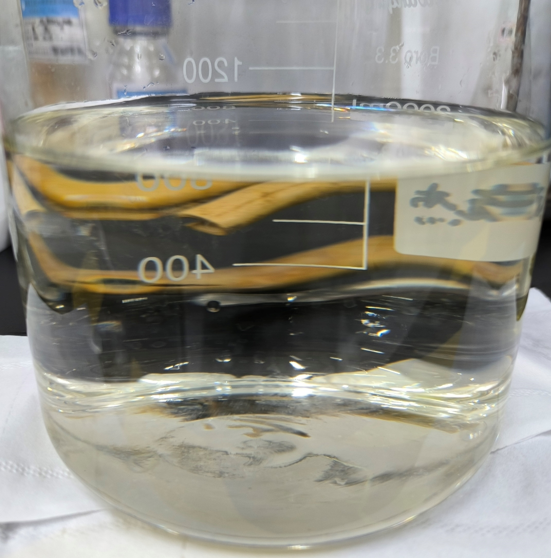


(a) (b) (c)

Figure S5. Sample photos of each operation during the pretreatment process. (a) Nylon 54 salt fermentation broth. (b) Permeate for membrane separation (c) Filtrate for decolorization of activated carbon.





Figure S6. Linear fitting of the Clapeyron-Clausius equation for D150.





Figure S7. Linear fitting of the Clapeyron-Clausius equation for D315.





Figure S8.  Effect of flow rate on dynamic adsorption of D150.





Figure S9.  Effect of flow rate on dynamic adsorption of D315.
